# Supplementary material for: Open fire exposure increases the risk of pregnancy loss in South Asia
Source: Nat Commun. 2021 May 28;12:3205. doi: 10.1038/s41467-021-23529-7 (PMC8163851; doi:10.1038/s41467-021-23529-7)
Supplement: Supplementary file 1 — Supplementary Information [file 41467_2021_23529_MOESM1_ESM.pdf]

# Supplementary information of

*Open fire exposure increased the risk of pregnancy loss in South Asia*

Tao Xue[1,*,†], Guannan Geng[2,†], Yiqun Han[3], Huiyu Wang[1], Jiajianghui Li[1], Hong-tian Li[1], Yubo Zhou[1], Tong Zhu[4,*]

1. Institute of Reproductive and Child Health / Ministry of Health Key Laboratory of Reproductive Health and Department of Epidemiology and Biostatistics, School of Public Health, Peking University Health Science Centre, Beijing, China, 100191
2. School of Environment, Tsinghua University, Beijing, China, 100085
3. Environmental Research Group, MRC Centre for Environment and Health, Imperial College London, London, UK
4. BIC-ESAT and SKL-ESPC, College of Environmental Science and Engineering, Peking University Beijing, China, 100871

† The authors equality contributed to the paper.

* Correspond to Dr. Tao Xue, txue@hsc.pku.edu.cn; Dr. Tong Zhu, tzhu@pku.edu.cn

**Supplemental Tables**

Supplementary Table 1 Population characteristics of the analyzed samples.

| Variable | Group | Pregnancy loss | Miscarriage | Stillbirth |
|---|---|---|---|---|
| | | Samples (N) | | |
| Matched number of controls | 1 | 10328 | 8075 | 2253 |
| | 2 | 8005 | 6537 | 1468 |
| | 3 | 3724 | 2940 | 784 |
| | 4 | 1720 | 1329 | 391 |
| | ⩾5 | 1099 | 849 | 250 |
| Country | Bangladesh | 2864 | 2083 | 781 |
| | India | 18862 | 15386 | 3476 |
| | Pakistan | 3150 | 2261 | 889 |
| Education | No education | 7080 | 5068 | 2012 |
| | Primary | 3947 | 2971 | 976 |
| | Secondary or higher | 13849 | 11691 | 2158 |
| Residence* | Urban | 8162 | 6807 | 1355 |
| | Rural | 16714 | 12923 | 3791 |
| Insurance | Not covered | 18787 | 15115 | 3672 |
| | Covered | 2609 | 2124 | 485 |
| | Unknown | 3480 | 2491 | 989 |
| Employment status | Rare or never | 1434 | 1121 | 313 |
| | Occasional | 532 | 388 | 144 |
| | Seasonal | 201 | 148 | 53 |
| | All year | 6722 | 5127 | 1595 |
| | Unknown | 15987 | 12946 | 3041 |
| Anemia | No | 9198 | 7638 | 1560 |
| | Yes | 9788 | 7822 | 1966 |
| | Unknown | 5890 | 4270 | 1620 |
| Body mass index | Underweight | 4259 | 3227 | 1032 |
| | Normal | 13153 | 10580 | 2573 |
| | Overweight or obese | 4975 | 4128 | 847 |
| | Unknown | 2489 | 1795 | 694 |
| Smoking | No | 2594 | 2102 | 492 |
| | Yes | 18802 | 15137 | 3665 |
| | Unknown | 3480 | 2491 | 989 |
| | | Mean (standard deviation) | | |
| Fire $PM_{2.5}$ (μg/m$^3$) | Case | 1.30 (2.41) | 1.35 (2.60) | 1.11 (1.49) |
| | Control | 1.14 (1.34) | 1.17 (1.35) | 1.03 (1.28) |
| Total $PM_{2.5}$ (μg/m$^3$) | Case | 53.24 (26.57) | 53.28 (27.50) | 53.09 (22.67) |
| | Control | 53.19 (25.32) | 53.30 (25.92) | 52.76 (22.89) |

| | | | | |
|---|---|---|---|---|
| Fire emission (g dry-matter/m$^3$/month) | Case | 0.98 (8.37) | 1.10 (9.32) | 0.54 (2.33) |
| | Control | 0.80 (4.10) | 0.85 (4.34) | 0.59 (2.97) |
| Satellite burned area (%) | Case | 0.14 (1.83) | 0.15 (1.97) | 0.09 (1.18) |
| | Control | 0.09 (1.48) | 0.10 (1.52) | 0.07 (1.29) |
| Temperature (K) | Case | 297.12 (6.31) | 297.07 (6.61) | 297.29 (5.01) |
| | Control | 296.98 (6.50) | 296.92 (6.73) | 297.19 (5.55) |
| Humidity (g/kg) | Case | 11.12 (4.79) | 11.07 (5.05) | 11.34 (3.60) |
| | Control | 10.99 (4.93) | 10.99 (5.19) | 10.96 (3.75) |
| Age (years) | Case | 26.15 (5.76) | 26.31 (5.74) | 25.55 (5.79) |
| | Control | 24.48 (5.06) | 24.49 (5.03) | 24.45 (5.17) |

* In DHS surveys, the definition of urban-rural is questionnaire-based and country-specific.

Supplementary Table 2 Response rates for the surveys incorporated into this study.

| Country | DHS phase* | Response rate of the eligible woman (%) | | |
|---|---|---|---|---|
| | | Urban | Rural | Total |
| India | 7[1] | 95.8 | 97.0 | 96.7 |
| Pakistan | 5[2] | 93.3 | 95.3 | 94.5 |
| | 7[3] | 93.2 | 95.3 | 94.3 |
| Bangladesh | 4[4] | 98.3 | 98.8 | 98.6 |
| | 5[5] | 98.1 | 98.5 | 98.4 |
| | 6[6] | 97.0 | 98.4 | 97.9 |
| | 7[7] | 97.5 | 98.1 | 97.9 |

* The rates are collected from the following sources:

1. International Institute for Population Sciences - IIPS/India, ICF. India National Family Health Survey NFHS-4 2015-16.). IIPS and ICF (2017).

2. National Institute of Population Studies - NIPS/Pakistan, Macro International. Pakistan Demographic and Health Survey 2006-07.). NIPS/Pakistan and Macro International (2008).

3. National Institute of Population Studies - NIPS/Pakistan, ICF. Pakistan Demographic and Health Survey 2017-18.). NIPS/Pakistan and ICF (2019).

4. National Institute of Population Research Training - NIPORT/Bangladesh, Mitra, Associates/Bangladesh, ORC Macro. Bangladesh Demographic and Health Survey 2004.). NIPORT, Mitra and Associates, and ORC Macro (2005).

5. National Institute of Population Research Training - NIPORT/Bangladesh, Mitra, Associates/Bangladesh, Macro International. Bangladesh Demographic and Health Survey 2007.). NIPORT, Mitra and Associates, and Macro International (2009).

6. National Institute of Population Research Training - NIPORT/Bangladesh, Mitra, Associates/Bangladesh, ICF International. Bangladesh Demographic and Health Survey 2011.). NIPORT, Mitra and Associates, and ICF International (2013).

7. National Institute of Population Research Training - NIPORT/Bangladesh, Mitra, Associates, ICF International. Bangladesh Demographic and Health Survey 2014.). NIPORT, Mitra and Associates, and ICF International (2016).

Supplementary Table 3 Comparison between the GEOS-Chem simulations and the satellite-based $PM_{2.5}$ concentrations.

| Year | Mean bias[#] (μg/m$^3$) | Root-squared-mean error (μg/m$^3$) | Regression* | | Correlation $R^2$ [†] |
|---|---|---|---|---|---|
| | | | Intercept | Slope | |
| 2000 | 7.64 | 14.09 | 5.99 | 1.10 | 0.53 |
| 2001 | 10.43 | 16.90 | 9.84 | 1.03 | 0.47 |
| 2002 | 10.05 | 16.31 | 9.53 | 1.03 | 0.50 |
| 2003 | 10.78 | 17.38 | 9.92 | 1.05 | 0.50 |
| 2004 | 9.68 | 16.52 | 10.64 | 0.95 | 0.51 |
| 2005 | 9.04 | 15.00 | 11.29 | 0.88 | 0.50 |
| 2006 | 8.67 | 15.74 | 11.94 | 0.84 | 0.48 |
| 2007 | 8.03 | 15.18 | 11.59 | 0.83 | 0.51 |
| 2008 | 10.48 | 17.20 | 14.61 | 0.82 | 0.55 |
| 2009 | 7.46 | 14.07 | 12.26 | 0.79 | 0.60 |
| 2010 | 8.43 | 16.21 | 12.40 | 0.82 | 0.52 |
| 2011 | 6.25 | 16.07 | 13.69 | 0.68 | 0.49 |
| 2012 | 6.43 | 14.54 | 13.30 | 0.71 | 0.54 |
| 2013 | 8.23 | 15.99 | 14.79 | 0.73 | 0.53 |
| 2014 | 6.84 | 14.37 | 13.57 | 0.74 | 0.60 |

* The regression is performed as the GEOS-chem simulations against the satellite-based $PM_{2.5}$ concentrations.

# The mean bias is calculated as the difference between the satellite-based $PM_{2.5}$ concentrations and the GEOS-chem simulations. In other words, in the comparison, the satellite-based $PM_{2.5}$ concentrations are treated as the reference values.

[†] The correlation coefficients are calculated using Pearson's $R^2$.

## Supplemental Figures

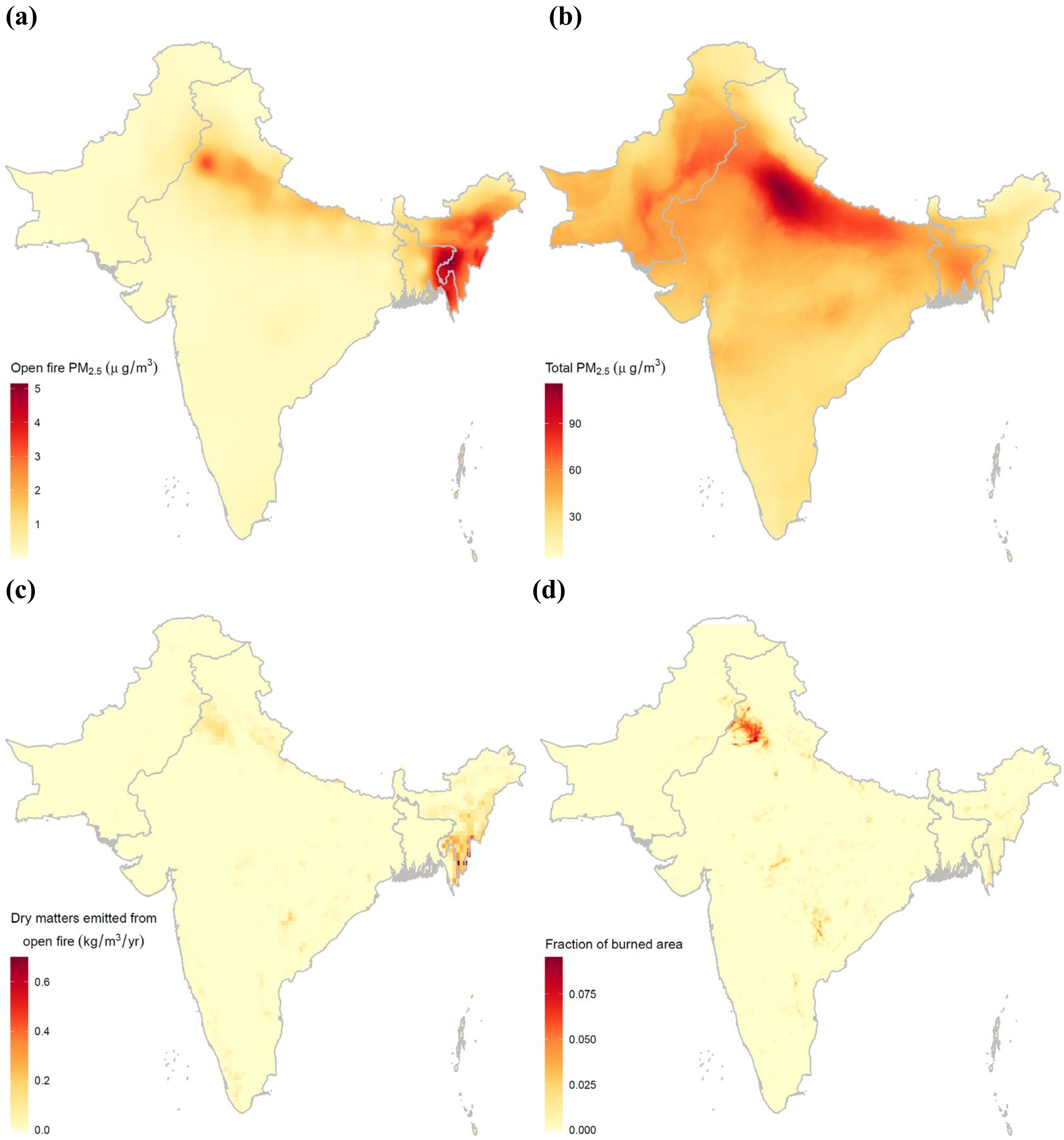


Supplementary Figure 1 Spatial distributions of exposure indicators, averaged during 2000-2014. (a) $PM_{2.5}$ attributable to open fire; (b) total $PM_{2.5}$; (c) fire emissions and (4) satellite remote sensing of burned area. The maps are generated by the corresponding author (T.X.) using the data from Natural Earth (https://www.naturalearthdata.com/). $PM_{2.5}$: particulate matter with a diameter of less than 2.5 μm.

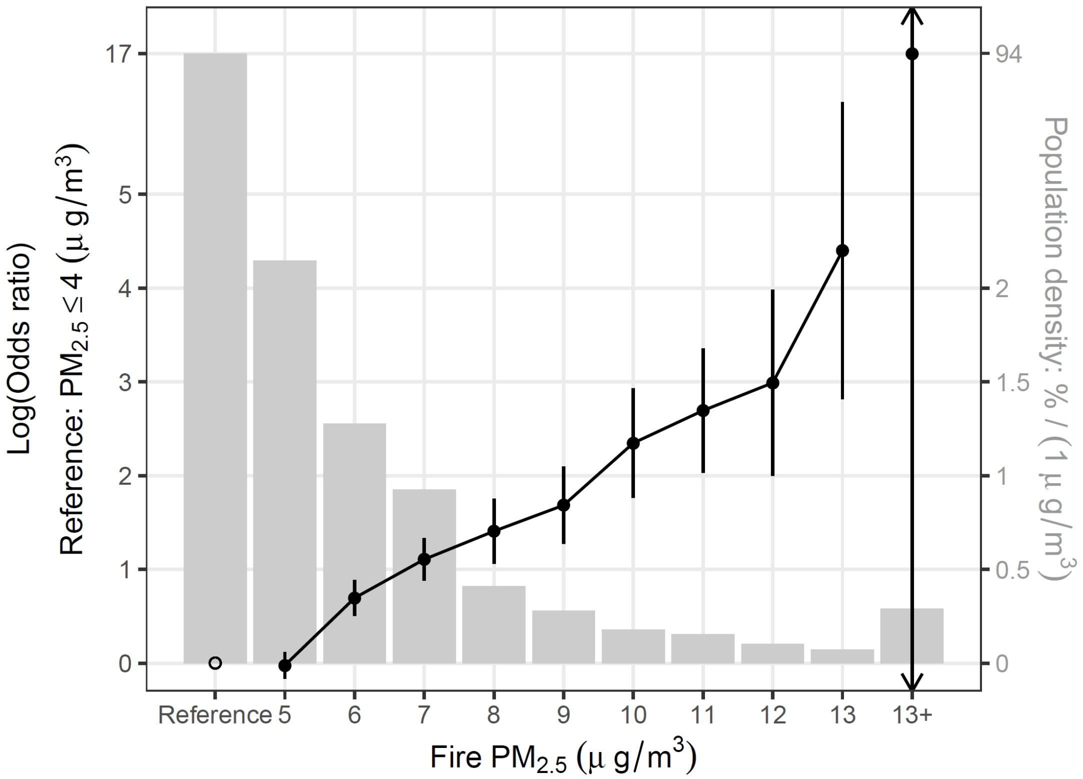


Supplementary Figure 2 The estimated association between the categorical variable of open fire $PM_{2.5}$ and pregnancy loss. The dots denote point-estimates of the association, the error bars denote the corresponding 95% confidence intervals, and the histograms (bars) denote the distribution of population density. All estimates are derived from the full-adjusted model with 24,876 independent mothers. $PM_{2.5}$: particulate matter with a diameter of less than 2.5 μm.

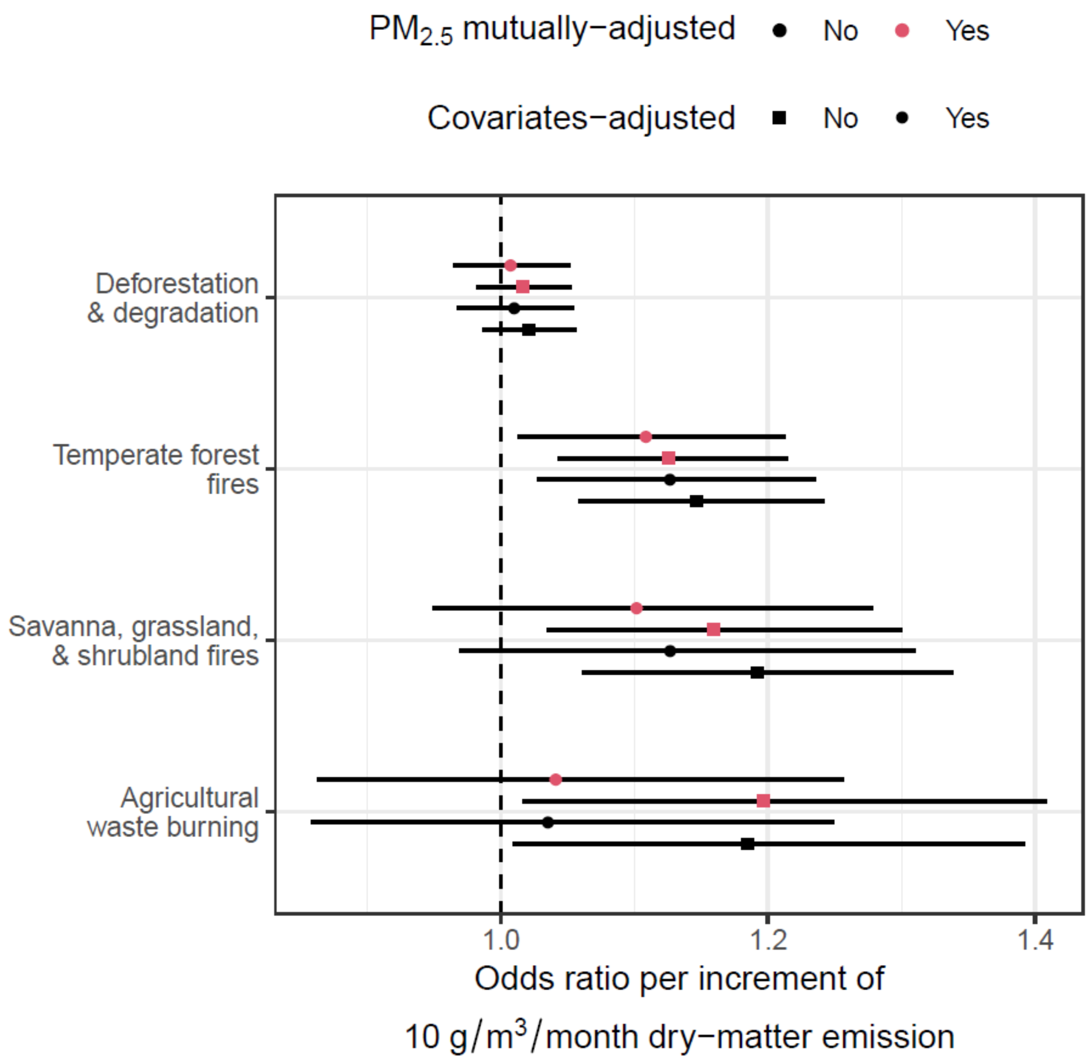


Supplementary Figure 3 The estimated associations between fire emissions and pregnancy loss by specific types of fire. The dots denote point-estimates of the associations, and the error bars denote the corresponding 95% confidence intervals. Each type of points (specified by colors and shapes) denotes a separate model with 24,876 independent mothers. $PM_{2.5}$: particulate matter with a diameter of less than 2.5 µm.

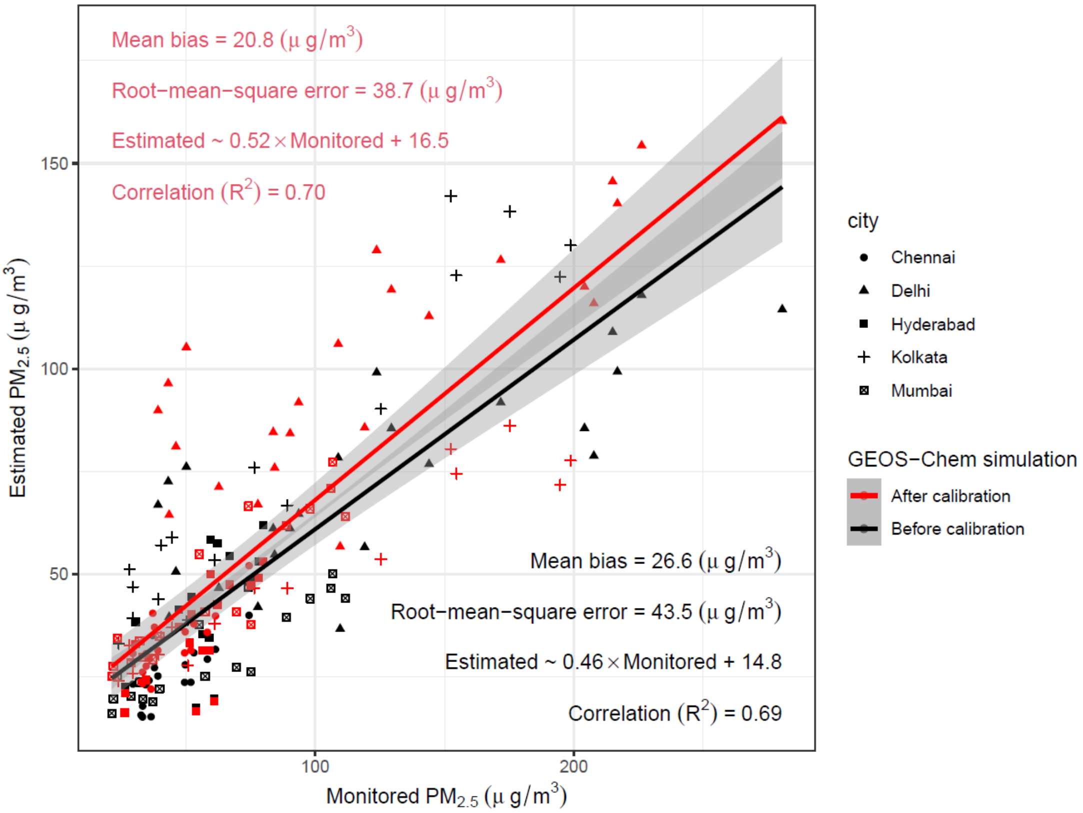


Supplementary Figure 4 Evaluation results for the GEOS-Chem simulated $PM_{2.5}$ concentrations in five Indian cities and monthly scale. The mean bias was calculated by the following equation: mean bias = monitored $PM_{2.5}$ – estimated $PM_{2.5}$. The correlation coefficient is calculated as the Pearson's $R^2$. The solid lines denote regressions of the estimates against the monitored values, and the grey ribbons denote their corresponding 95% confidence intervals. $PM_{2.5}$: particulate matter with a diameter of less than 2.5 μm.

**Supplementary codes: original R codes for epidemiological analyses**

1. R codes for models by different covariate adjustments and exposure indicators

```
load("Lng Fire and PregLoss.RData") # the data is obtained and manually assembled from multiple surveys from DHS as described in the main-text. Those surveys should be downloaded directly from DHS website. We cannot re-distribute them.

library(splines)
library(survival)

xs=c("FirePM","FireSat","FireEms","FireEms_AGRI","FireEms_DEFO","FireEms_SAVA","FireEms_TEMF")
lng$PM25gc=lng$PM25gc-lng$FirePM
fs<-c("end~x+strata(v2id)",
      "end~x+PM25gc+strata(v2id)",
"end~x+ns(TMP,3)+age+ns(HTY,3)+ns(bgn.m,4)+as.character(bgn.year)+strata(v2id)",
"end~x+PM25gc+ns(TMP,3)+age+ns(HTY,3)+ns(bgn.m,4)+as.character(bgn.year)+strata(v2id)")
names(fs)=c("unadjusted","PM25-unadjusted","adjusted","PM25-adjusted")

for(x in xs)
{
  lng$x=lng[,x]
  for(f in fs)
  {
    m<-clogit(as.formula(f),data=lng,iter.max=5000)
    tmpr=rbind(cbind(as.data.frame(summary(m)$coef)[1,]))
    tmpr$model=names(which(fs==f))
    tmpr$x=x
    if(f==fs[1]&x==xs[1]) coef=tmpr else coef=rbind(coef,tmpr)
  }
}
coef$OR=exp(coef$coef*coef$unit)
coef$lo=exp(coef$coef-coef$`se(coef)`*1.96)
coef$up= exp(coef$coef+coef$`se(coef)`*1.96)

coef # The model outputs
```

2. R codes for models by modifiers

```
load("Lng Fire and PregLoss.RData") # the data is obtained and manually assembled from multiple surveys from DHS as described in the main-text. Those surveys should be downloaded directly from DHS website. We cannot re-distribute them.

library(splines)
library(survival)

xs=c("FirePM","FireSat","FireEms","FireEms_AGRI","FireEms_DEFO","FireEms_SAVA","FireEms_TEMF")
lng$PM25gc=lng$PM25gc-lng$FirePM

f<-"end~ef:x+PM25gc+ns(TMP,3)+age+ns(HTY,3)+ns(bgn.m,4)+as.character(bgn.year)+strata(v2id)"
fb<-"end~x+PM25gc+ns(TMP,3)+age+ns(HTY,3)+ns(bgn.m,4)+as.character(bgn.year)+strata(v2id)"
efs=c("edu","urban","insurance","job","age","anemia","obesity","smoke","marry","gapc")
for(x in xs[1:3])
{
  lng$x=lng[,x]
  for(ef in efs)
  {
    tmp<-subset(lng,v2id%in%subset(lng,!is.na(ef)&end==1)$v2id)
    m<-clogit(as.formula(f),data=tmp,iter.max=5000)
    b<-clogit(as.formula(fb),data=tmp,iter.max=5000)
    tab<-as.data.frame(summary(m)$coef)
    tab<-tab[grepl(":x",rownames(tab)),]
    ids<-gsub(":x","",fixed=T,substr(rownames(tab),start=3,stop=50))
tmpr=cbind(x=x,modifier=ef,grp=ids,grp.n=as.numeric(table(tmp$ef)[ids]),grp.p=as.numeric(table(subset(tmp,end==1)$ef)[ids]),tab,LRpvalue=as.numeric(anova(m,b)$"P(>|Chi|)"[2]))
    if(ef==efs[1]&x==xs[1]) coef=tmpr else coef=rbind(coef,tmpr)
  }
}
coef$OR=exp(coef$coef*coef$unit)
coef$lo=exp(coef$coef-coef$`se(coef)`*1.96)
coef$up= exp(coef$coef+coef$`se(coef)`*1.96)

coef # The model outputs
```

3. R codes for nonlinear associations

```
load("Lng Fire and PregLoss.RData") # the data is obtained and manually assembled from multiple surveys from DHS as described in the main-text. Those surveys should be downloaded directly from DHS website. We cannot re-distribute them.

library(splines)
library(survival)

lng$PM25gc=lng$PM25gc-lng$FirePM

f<-"end~pspline(x)+PM25gc+ns(TMP,3)+age+ns(HTY,3)+ns(bgn.m,4)+as.character(bgn.year) + strata(v2id)"
for(x in c("FirePM”))
{
  lng$x=lng[,x]
  ERF=data.frame(x=seq(0,quantile(lng$x,0.999),length=100),PM25gc=0,TMP=0,
age=unique(lng$age)[1],HTY=0,bgn.m=1,bgn.year=2000,v2id=unique(lng$v2id)[1])
  m<-clogit(as.formula(f),data=lng,iter.max=5000)
  tmp=predict(m,newdata = ERF,type="terms",se=T)

  ERF$y=tmp$fit[,1]
  ERF$se=tmp$se.fit[,1]
  ERF$type=x
  if(x=="FirePM") ERFs=ERF else ERFs=rbind(ERFs,ERF)
}

head(ERF) # The model outputs for nonlinear association

x="FirePM"
lng$x=lng[,x]
lng$x2=cut(lng$x,c(-Inf,4:13,Inf))
f<-"end~x2+PM25gc+ns(TMP,3)+age+ns(HTY,3)+ns(bgn.m,4)+as.character(bgn.year)+strata(v2id)"
m<-clogit(as.formula(f),data=lng,iter.max=5000)

summary(m) # The model outputs for association by categorical variable
```
