# Supplementary material for: Open fire exposure increases the risk of pregnancy loss in South Asia
Source: Nat Commun. 2021 May 28;12:3205. doi: 10.1038/s41467-021-23529-7 (PMC8163851; doi:10.1038/s41467-021-23529-7)
Supplement: Supplementary file 3 — Reporting Summary [file 41467_2021_23529_MOESM3_ESM.pdf]

nature research

Corresponding author(s): Tao Xue; Tong Zhu

Last updated by author(s): Feb 27, 2021

Reporting Summary

Nature Research wishes to improve the reproducibility of the work that we publish. This form provides structure for consistency and transparency in reporting. For further information on Nature Research policies, see our Editorial Policies and the Editorial Policy Checklist.

Statistics

For all statistical analyses, confirm that the following items are present in the figure legend, table legend, main text, or Methods section.

n/a	Confirmed	
☐	☒	The exact sample size (n) for each experimental group/condition, given as a discrete number and unit of measurement
☐	☒	A statement on whether measurements were taken from distinct samples or whether the same sample was measured repeatedly
☐	☒	The statistical test(s) used AND whether they are one- or two-sided<br>*Only common tests should be described solely by name; describe more complex techniques in the Methods section.*
☐	☒	A description of all covariates tested
☒	☐	A description of any assumptions or corrections, such as tests of normality and adjustment for multiple comparisons
☐	☒	A full description of the statistical parameters including central tendency (e.g. means) or other basic estimates (e.g. regression coefficient) AND variation (e.g. standard deviation) or associated estimates of uncertainty (e.g. confidence intervals)
☐	☒	For null hypothesis testing, the test statistic (e.g. F, t, r) with confidence intervals, effect sizes, degrees of freedom and P value noted<br>*Give P values as exact values whenever suitable.*
☒	☐	For Bayesian analysis, information on the choice of priors and Markov chain Monte Carlo settings
☒	☐	For hierarchical and complex designs, identification of the appropriate level for tests and full reporting of outcomes
☐	☒	Estimates of effect sizes (e.g. Cohen's d, Pearson's r), indicating how they were calculated

Our web collection on statistics for biologists contains articles on many of the points above.

Software and code

Policy information about availability of computer code

Data collection	The data were directly downloaded from open sources. No software or code was involved in data collection.
Data analysis	The exposure data of fire-sourced PM2.5 were generated using standard GEOS-Chem model (version 11-01), which can be obtained from http://acmg.seas.harvard.edu/geos/. The statistical data were analyzed using R (version 4.0.2). The statistical inference on the conditional logit model was performed using the R package survival. The codes to generate Figures are within the supplemental materials.

For manuscripts utilizing custom algorithms or software that are central to the research but not yet described in published literature, software must be made available to editors and reviewers. We strongly encourage code deposition in a community repository (e.g. GitHub). See the Nature Research guidelines for submitting code & software for further information.

Data

Policy information about availability of data

All manuscripts must include a data availability statement. This statement should provide the following information, where applicable:

- Accession codes, unique identifiers, or web links for publicly available datasets
- A list of figures that have associated raw data
- A description of any restrictions on data availability

The Demographic and Health Survey (DHS) data, satellite fire data (MODIS MCD64A1), global fire emission database (GFED, version 4), anthropogenic emission inventory of Community Emissions Data System (CEDS), Modern-Era Retrospective analysis for Research and Applications Version 2 (MERRA-2) data, and satellite-based PM2.5 data that support the findings of this study are available from https://www.dhsprogram.com/, https://lpdaacsvc.cr.usgs.gov/appeears/, http://globalfiredata.org/, https://esgf-node.llnl.gov/search/input4mips/, https://disc.gsfc.nasa.gov/, and http://fizz.phys.dal.ca/~atmos/martin/?page_id=140, respectively. The fire PM2.5 data that supporting the findings of this study are derived from the GFED, CEDS, MERRA-2 and satellite-based PM2.5 data using a open-sourced GEOS-Chem model (http://acmg.seas.harvard.edu/geos/). The specific simulations are available from the corresponding author upon reasonable request.

The ground-surface monitoring data to evaluate GEOS-Chem simulations are from https://in.usembassy.gov/embassy-consulates/new-delhi/air-quality-data/. The derived data to reproduce the Figures are within the supplemental files.

Field-specific reporting

Please select the one below that is the best fit for your research. If you are not sure, read the appropriate sections before making your selection.

☐ Life sciences ☐ Behavioural & social sciences ☒ Ecological, evolutionary & environmental sciences

For a reference copy of the document with all sections, see nature.com/documents/nr-reporting-summary-flat.pdf

Ecological, evolutionary & environmental sciences study design

All studies must disclose on these points even when the disclosure is negative.

Study description	We conducted a self-comparison case-control study to look at the association between fire smoke exposure and pregnancy loss in South Asia. Each eligible participant reported a case of pregnancy loss and at least one normal delivery as the corresponding control. Maternal exposure to fire smoke was assessed primarily using a chemical transport model as the concentrations of PM2.5 attributable to open fires. Statistical inference was based on the difference between fire exposure level at pregnancy loss and that at normal delivery, within the same mother.
Research sample	The study was based on extant database of demographic and health survey (DHS). Since our exposure data were available from 2000 to 2014, we collected all available samples in DHS surveys from the three largest South Asia countries, India, Pakistan, and Bangladesh. All eligible samples were finally involved in our study. This study finally 24,876 mothers from seven geocoded DHS surveys including four in Bangladesh (DHS phases 4–7), two in Pakistan (phase 5 and 7), and one in India (phase 7). All the surveys can be publicly obtained from DHS website: https://dhsprogram.com/.
Sampling strategy	This study was based on extant DHS database. From all samples recorded in geocoded DHS surveys, we selected all mothers who reported a case of pregnancy loss and at least one successful delivery. No further sampling procedure was conducted.
Data collection	The DHS surveys are household-based instruments, and the samples were selected using a complex two-stage design. For each national survey, in the first stage, enumeration areas are selected according to census data; in the second stage, households are sampled from an updated list of households. The females of reproductive age (15–49 years) in each household were of particular interest, and their records for socioeconomic status, fertility, reproductive history, infant mortality, etc. were screened by well-trained interviewers using standard questionnaires. DHS utilized a team approach to data collection. Usually, each DHS team is composed of a supervisor, field editor, and several interviewers. The cases are recorded by the interviewers using the reproductive module of the uniform questionnaire. The geographic information are recorded by the Global Position System (GPS) device. For eligible female participates, the response rate was varied by countries and ranged from 93-99% for the incorporated surveys.
Timing and spatial scale	Since the key datasets related to exposure assessments (e.g., CEDS inventory, 1750-2014; and MODIS burned areas, 2000-present) are simultaneously available from 2000 to 2014, we included all eligible samples during the same period (i.e., 2000-2014) from seven geocoded DHS surveys including four in Bangladesh (DHS phases 4–7), two in Pakistan (phase 5 and 7), and one in India (phase 7) were analyzed.
Data exclusions	According to this epidemiological design, we only selected mothers who reported a case of pregnancy loss and at least one successful delivery. Of the 782,918 female respondents to the seven surveys, 102,427 were cases of pregnancy loss. Of these, 31,303 losses occurred within our study period, i.e., 2000–2014. After excluding 6,427 cases who could not be matched with eligible controls (i.e., successful deliveries with valid exposure assessments), the analysis finally involved 24,876 mothers. In total, they reported 75,262 delivery events, including the most recent case and all available controls for each mother.
Reproducibility	All attempts to repeat the experiment were successful.
Randomization	NA. This study is an observational study, and we didn't perform further sampling or randomizing.
Blinding	This study is an observational, and thus is not applicable for a blinding design.

Did the study involve field work? ☐ Yes ☒ No

Reporting for specific materials, systems and methods

We require information from authors about some types of materials, experimental systems and methods used in many studies. Here, indicate whether each material, system or method listed is relevant to your study. If you are not sure if a list item applies to your research, read the appropriate section before selecting a response.

Materials & experimental systems

n/a	Involved in the study
☒	☐ Antibodies
☒	☐ Eukaryotic cell lines
☒	☐ Palaeontology and archaeology
☒	☐ Animals and other organisms
☐	☒ Human research participants
☒	☐ Clinical data
☒	☐ Dual use research of concern

Methods

n/a	Involved in the study
☒	☐ ChIP-seq
☒	☐ Flow cytometry
☒	☐ MRI-based neuroimaging

Human research participants

Policy information about studies involving human research participants

Population characteristics	This study examined 24,876 cases of pregnancy loss and 50,386 matched controls (normal deliveries) during 2000–2014. Of these, 11.5% were in Bangladesh, 12.7% in Pakistan, and the rest were in India. The mean age at pregnancy loss was 26.15 years (standard deviation [SD] = 5.76 years), which was older than that at normal delivery (mean = 24.48 years; SD = 5.06 years).
Recruitment	This study is an observational study based on the extent data collected by DHS fieldworkers. We utilized all available samples from the DHS databases. We didn't further selected the samples.
Ethics oversight	NA. This study is based on publicly-available population data. We adhered to the DHS data usage guidelines in all our analyses. No further ethic approavment is required.

Note that full information on the approval of the study protocol must also be provided in the manuscript.
