# Supplementary material for: Open fire exposure increases the risk of pregnancy loss in South Asia
Source: Nat Commun. 2021 May 28;12:3205. doi: 10.1038/s41467-021-23529-7 (PMC8163851; doi:10.1038/s41467-021-23529-7)
Supplement: Supplementary file 4 — Source Data [file 41467_2021_23529_MOESM4_ESM.zip › Supplemental-Codes.html]

Supplemental Codes to reproduce Figures 1-5 for the manuscript of ‘Open fire exposure increases the risk of pregnancy loss in South Asia’


# Supplemental Codes to reproduce Figures 1-5 for the manuscript of ‘Open fire exposure increases the risk of pregnancy loss in South Asia’

#### Tao Xue, et al.

#### 2020/12/29

## Figure 1a

The codes are utilized to reproduce Figure 1(a). Please load the ‘Fig 1a Data.RData’ before running the following R codes. The data contain the following datasets.

1. *fire*: mean concentration of open fire PM2.5 during 2000–2014;
2. *GPS*: locations of surveyed samples; The dataset dosn’t contain the true DHS surveyed locations, but the mimic values generated by the authors. The true DHS locations can be obtained from https://www.dhsprogram.com/.
3. *line1*, *line2*, and *line3*: the geographic boundaries for the three countries involved in this study. The authors are neutral on issues of disputed regions and borders.

```
load("Fig 1a Data.RData")
library(ggplot2)
library(openair)
library(sp)
ggplot(data=fire)+
  geom_tile(aes(x=lon,y=lat,fill=val))+
  geom_point(data=GPS,aes(x=lon,y=lat),col=1,shape=4,size=0.005,alpha=0.2)+
  geom_polygon(data=line1,aes(x=long,y=lat,group=group),col="grey",alpha=0,fill=NA,size=0.5)+
  geom_text(data=data.frame(x=coordinates(line1)[,1],
                            y=coordinates(line1)[,2],
                            lab1="Bangladesh"),aes(x=x,y=y,label=lab1),col="blue",size=10)+
  geom_polygon(data=line2,aes(x=long,y=lat,group=group),col="grey",alpha=0,fill=NA,size=0.5)+
  geom_text(data=data.frame(x=coordinates(line2)[,1],
                            y=coordinates(line2)[,2],
                            lab1="Pakistan"),aes(x=x,y=y,label=lab1),col="blue",size=10)+
  geom_polygon(data=line3,aes(x=long,y=lat,group=group),col="grey",alpha=0,fill=NA,size=0.5)+
  geom_text(data=data.frame(x=coordinates(line3)[,1],
                            y=coordinates(line3)[,2],
                            lab1="India"),aes(x=x,y=y,label=lab1),col="blue",size=10)+
  theme_void()+
  scale_fill_gradientn(colors=openColours("heat",50),name=expression('Open fire'~PM[2.5]~(mu~g/m^3)))+
  guides(fill = guide_colorbar(nbin = 100,barheight=10,barwidth=1,order=1))+
  theme(legend.position = c(0.01,0.01),
        legend.justification = c(0,0),
        legend.box = "horizontal",
        legend.box.just = "bottom")+
  scale_x_continuous(expand = c(0,0))+
  scale_y_continuous(expand = c(0,0))
```

## Figure 1b

The codes are utilized to reproduce Figure 1(b). Please load the ‘Fig 1a Data.RData’ before running the following R codes. The data contain a dataset named as *ts*, which has the variable of population-weighted concentration of fire PM2.5 (*FirePM*) and of total PM2.5 (*PM25*) by month.

```
load("Fig 1b Data.RData")
library(ggplot2)
library(chron)
ggplot(data=ts)+
  geom_area(aes(x=date,y=PM25,fill="A"),alpha=0.6)+
  geom_area(aes(x=date,y=FirePM,fill="B"),alpha=0.6)+
  geom_path(aes(x=date,y=PM25),col=1)+
  geom_path(aes(x=date,y=FirePM),col=1)+
  geom_point(aes(x=date,y=PM25,col=season))+
  geom_point(aes(x=date,y=FirePM,col=season))+
  scale_y_sqrt(breaks=seq(5,75,10),name=expression(PM[2.5]~(mu~g/m^3)))+
  scale_x_chron(format="%Y",breaks=subset(ts,month%in%c(1))$date,name="Year")+
  scale_fill_manual(values=c("grey90","grey60"),labels=expression("Non-fire PM"[2.5],"Fire PM"[2.5]))+
  theme_bw()+
  theme(legend.title = element_blank(),
        legend.position=c(0.01,1),
        legend.justification = c(0,1),
        legend.background = element_blank(),
        panel.grid.minor = element_blank(),
        legend.box = "horizontal")+
  guides(color=guide_legend(ncol=4))
```

## Figure 2

The codes are utilized to reproduce Figure 2. Please load the ‘Fig 2 Data.RData’ before running the following R codes. The data contain (1) a dataset named as *associations\_modifier*, which contains the estimated odds by different subpopulation indicators, and (2) a dataset named as *labs*, which contains the p-values for the corresponding likelihood ratio tests.

```
load("Fig 2 Data.RData")
library(ggplot2)
ggplot(data=associations_modifier)+
  geom_linerange(aes(x=label,ymin=lo,ymax=up))+
  geom_point(aes(x=label,y=OR))+
  facet_wrap(~modifier,scales="free_y",ncol=1,strip.position = "left")+
  coord_flip()+
  theme_bw()+
  theme(axis.title.y = element_blank(),
        strip.text.y = element_text(angle=180))+
  ylab(expression("Odds ratio per increment of"~1~mu~g/m^3~fire~PM[2.5]))+
  geom_text(data=labs,aes(x=x,y=max(y),label=pval),hjust=1,vjust=0)+
  geom_hline(aes(yintercept=1),linetype=2)
```

## Figure 3

The codes are utilized to reproduce Figure 3. Please load the ‘Fig 3 Data.RData’ before running the following R codes. The data contain a dataset named as *associations\_subset*, which contains the estimated odds ratios from different subsets of the whole sample, according to the sensitivity analyses.

```
load("Fig 3 Data.RData")
library(ggplot2)
ggplot(data=associations_subset)+
  geom_linerange(aes(x=label,ymin=lo,ymax=up,group=paste(PM25adj,covadj)),position = position_dodge(width=0.5))+
  geom_point(aes(x=label,y=OR,color=PM25adj,shape=covadj),position = position_dodge(width=0.5))+
  coord_flip()+
  theme_bw()+
  theme(axis.title.y = element_blank(),
        strip.text.y = element_text(angle=180),
        legend.position = "top",legend.direction = "horizontal",legend.box = "horizontal",
        legend.spacing = unit(0.001,"mm"),
        legend.background = element_blank())+
  ylab(expression("Odds ratio per increment of"~1~mu~g/m^3~fire~PM[2.5]))+
  geom_hline(aes(yintercept=1),linetype=2)+
  scale_x_discrete(labels=parse(text=sort(unique(associations_subset$label))))+
  scale_shape_manual(values=c(15,16),name="Covariates-adjusted")+
  scale_color_manual(values=c(1,2),name=expression(PM[2.5]~"mutually-adjusted"))
```

## Figure 4

The codes are utilized to reproduce Figure 4. Please load the ‘Fig 4 Data.RData’ before running the following R codes. The data contain (1) a dataset named as *curve*, which contains the nonlinear association and (2) a dataset named as *freq*, which contains the distribution of studied population by exposure levels.

```
load("Fig 4 Data.RData")
library(ggplot2)
ggplot(data=curve)+
  geom_bar(data=freq,aes(x=x,y=sqrt(den)),stat="identity",fill="grey80")+
  geom_path(aes(x=x,y=(exp(y)-1)))+
  geom_path(aes(x=x,y=(exp(y-se*1.96)-1)),linetype=2)+
  geom_path(aes(x=x,y=(exp(y+se*1.96)-1)),linetype=2)+
  theme_bw()+ylab(expression("Odds ratio of pregnancy loss"))+
  xlab(expression(Fire~PM[2.5]~(mu~g/m^3)))+
  scale_x_continuous(breaks=seq(0,14,2))+
  scale_y_continuous(breaks=c(1,seq(5,45,5))-1,labels=c(1,seq(5,45,5)),sec.axis = dup_axis(~.,breaks=sqrt(seq(0,50,10)*5),labels=seq(0,50,10),name=expression('Population density: % /'~(1~mu~g/m^3))))+
  theme(axis.title.y.right = element_text(color="grey60"),
        axis.text.y.right = element_text(color="grey60"),
        panel.grid.minor = element_blank())
```

## Figure 5

The codes are utilized to reproduce Figure 5. Please load the ‘Fig 5 Data.RData’ before running the following R codes. The data contain (1) a dataset named as *contribution\_total*, which contains the estimates for the whole study domain, and (2) a dataset named *contribution\_country*, which contains the estimates by countries.

```
load("Fig 5 Data.RData")
library(ggplot2)
library(gridExtra)
Fig1<-ggplot(data=contribution_total)+
  geom_bar(aes(x=x,y=val),stat="identity",fill="grey80")+
  geom_linerange(aes(x=x,ymin=lo,ymax=up),col=1)+
  facet_wrap(~type,labeller = label_parsed,scale="free_y",strip.position = "left")+
  scale_x_continuous(breaks=1:2,labels=expression("Fire PM"[2.5],"Non-fire PM"[2.5]))+
  theme_classic()+
  theme(axis.title = element_blank())

Fig2<-ggplot(data=contribution_country)+
  geom_bar(aes(x=x,y=val),stat="identity",fill="grey80")+
  geom_linerange(aes(x=x,ymin=lo,ymax=up),col=1)+
  facet_grid(type~county,labeller = label_parsed,scale="free_y")+
  scale_x_continuous(breaks=1:2,labels=expression("Fire PM"[2.5],"Non-fire PM"[2.5]))+
  theme_classic()+
  theme(axis.title = element_blank())+
  geom_hline(aes(yintercept=0),col="grey80")
grid.arrange(Fig1,Fig2,ncol=1,heights=c(3,4.5))
```
